# Cytokine production of mononuclear leukocytes in response to respiratory syncytial virus is increased in COPD but suppressed in non-COPD tobacco smokers

**DOI:** 10.1186/s10020-025-01277-4

**Published:** 2025-06-12

**Authors:** Sarah D. Yanik, Kaschin Jamal Jameel, Simon Rohde, Paul Bürger, Eike Bülthoff, Thomas Grunwald, Juliane Kronsbein, Andrea Koch, Michael R. Edwards, Matthias Tenbusch, Jürgen Knobloch

**Affiliations:** 1 Medical Clinic III for Pneumology, Allergology, Sleep- and Respiratory Medicine, Bergmannsheil University Hospital, Ruhr-University Bochum, Bürkle-de-la-Camp-Platz 1, Bochum, 44789 Germany; 2 Fraunhofer Institute for Cell Therapy and Immunology, Perlickstr. 1, Leipzig, 04103 Germany; 3 Johannes Kepler Universität (JKU) und Kepler Universitätsklinikum (KUK) Linz; MC III, Innere Medizin 4, Pneumologie. Krankenhausstr. 9, Linz, 4020 Austria; 4 Ludwig-Maximilians-University of Munich (LMU) and DZL (German Center of Lung Science), Munich, 81377 Germany; 5 National Heart and Lung Institute, Imperial College, London, UK; 6 Institute of Clinical and Molecular Virology, University Hospital Erlangen, Friedrich- Alexander-Universität (FAU) Erlangen-Nürnberg, Schlossgarten 4, Erlangen, 91054 Germany; 7 FAU Profile Center Immunomedicine (FAU I-MED), Friedrich-Alexander-Universität (FAU), Erlangen-Nürnberg, Schlossplatz 1, Erlangen, D-91054 Germany

**Keywords:** RSV, COPD, Exacerbation, Circulating immune cells, Cytokine overproduction

## Abstract

**Background:**

Respiratory syncytial virus (RSV) induces exacerbations of chronic obstructive pulmonary disease (COPD) that are critical for disease progression and burden. COPD subjects have an increased susceptibility to viral respiratory infections. We aimed to identify underlying systemic immune pathologies that could be used as drug targets to reduce exacerbations.

**Methods:**

Peripheral blood mononuclear cells were isolated from 16 healthy never smokers, 17 current smokers without airflow limitation, and 17 COPD subjects. The cells were cultured and infected with RSV for 24 h or seven days. IFNα, T-cell- and inflammatory cytokines, the expression of interferon-stimulating genes (ISGs), and virus load in supernatants were measured by ELISA or real-time PCR, respectively. Data were compared between the three patient groups.

**Results:**

RSV induced CCL2, CCL5, IFNα, IFNγ, IL1-β, IL-6, IL-8, and TNFα but not IL-4, IL-5, IL-17, GM-CSF, and TGFβ. CCL2 was unchanged between the groups. All other cytokines were either increased or produced for a longer period of time in COPD but were reduced or not produced at all in smokers. Virus copy numbers were increased in COPD but reduced in smokers. RSV induced MxA, OAS, and Viperin expression with differences between the groups.

**Conclusion:**

Circulating immune cells in COPD might cause cytokine overproduction in response to RSV after recruitment to the site of infection and might contribute to the increase in inflammation in exacerbations. This might be explained by differences in RSV replication efficacy and ISG expression. We provide first indication for ISGs and circulating cells as drug targets to reduce or prevent exacerbations.

**Supplementary Information:**

The online version contains supplementary material available at 10.1186/s10020-025-01277-4.

## Background

Airway and systemic inflammation are central in the pathogenesis of cigarette smoke-induced chronic obstructive pulmonary disease (COPD) because they induce irreversible airway remodeling and co-morbidities. A large subgroup of COPD patients suffers from frequent exacerbations that come along with an increase in inflammation and result in an acute worsening of symptoms often leading to hospitalization. Exacerbations are a main trigger of disease progression and are mainly caused by infections with respiratory bacteria and viruses. Exacerbations that are triggered by respiratory viral infections are more severe and are associated with longer recovery times than those triggered by other factors (Wedzicha et al., 2007). What complicates matters is the fact that COPD patients have an increased susceptibility to viral airway infections compared to healthy subjects implicating defects in the appropriate immune responses (Frickmann et al. 2012; Linden et al. 2019; Mallia et al. 2011). Our goal of this presented study was to identify underlying immune pathologies and related potential drug targets.

Among the viruses that are most often found in COPD exacerbations is respiratory syncytial virus (RSV) (Frickmann et al. 2012; Wedzicha et al., 2007), a negative stranded RNA paramyxovirus that belongs to the family of *Pneumoviridae* (Russell et al. 2017). Data from animal studies indicate that RSV enhances inflammatory processes, airway remodeling and tissue destruction in cigarette smoke-induced COPD. Therefore, preventing RSV infection might have the potential to significantly impact COPD severity and progression (Foronjy et al. 2014). Despite recommendations to vaccinate patients with COPD, vaccination rates remain suboptimal (Simon et al. 2023) and an effective drug treatment is currently not available (Russell et al. 2017).

RSV infection induces an initial strong interferon (IFN) α and neutrophil response followed by a predominantly CD8 + and IFNγ-based T-cell response as well as by B cell activation finally leading to IgG antibody production. Upregulated Th1/Tc1 cytokines, such as IFNγ, are protective, the additionally observed Th2 responses might be rather deleterious (Russell et al. 2017). IFNα, IFNβ, and IFNγ induce the expression of various interferon stimulated genes (ISGs) to combat intracellular RSV. For example, both, IFNα and IFNγ, induce 2’,5’-oligoadenylate synthase (OAS) that blocks RSV replication via RNase L-mediated degradation of viral RNA (Behera et al. 2002; Silverman et al., 2007). IFNα further increases viperin to suppress RSV replication (Jumat et al. 2015; McGillivary et al. 2013). In contrast, RSV, particularly strain A2, might be resistant to the antiviral effects of IFNα-induced myxovirus resistant gene A (MxA) (Atreya et al., 1999), although this ISG is a blood cell marker for RSV infection at least in children (Halminen et al. 1997).

Interestingly, there is a partial overlap in the cytokines and chemokines that are usually upregulated in response to RSV infections and that are present in COPD systemic inflammation. This applies to CCL2, interleukin (IL)−1β, IL-6, IL-8, TNF, and IFNγ (Chen at al., 2021; Russell et al. 2017; Tkacova 2010). This leads us to assume that the inflammatory state of circulating leukocytes might interfere with the immune response to RSV in COPD. This idea is supported by previous studies that have demonstrated systemic defects in circulating CD4 + T-cells and in monocytes as a possible mechanistic cause of the increased susceptibility of COPD subjects to bacterial respiratory infections (Knobloch et al. 2011, 2016, 2019). To address this question, we have used a primary human cell culture model, the peripheral blood mononuclear cells (PBMCs). This model considers innate immune cells like monocytes, dendritic cells, and natural killer cells as well as T- and B-cells from the adaptive immunity and their interaction. In response to acute RSV infections in vivo, these circulating cells get recruited into the corresponding tissue and/or lymph nodes where they come in contact with the pathogen. Therefore, we infected the cultured PBMCs with RSV and compared their responses between healthy never smokers, current smokers without obstructive lung disease, and COPD subjects. We hypothesized to see differences in the cytokine response, replication efficiency, and expression of ISGs between cells of COPD subjects and healthy subjects indicating for systemic immune pathologies in COPD with relevance for local inflammation and associated exacerbations.

## Methods

### Sample size calculation

Sample size calculation was done with the Java program Piface (2023-06-14: https://homepage.divms.uiowa.edu/~rlenth/Power/), based on the following primary outcome: difference in virus induced cytokine release from PBMCs between healthy never-smokers (NS), current smokers without airway obstruction (S) and COPD. On the basis of preliminary experiments with *n* = 4 subjects of each group it was estimated that the sample size to achieve a power of 1-β = 0.8 for a One-way ANOVA on ranks test at α = 0.05 should be 13 subjects in each group. We increased the number of subjects to *n* = 17 for each cohort to compensate a putative loss of samples because of errors by performing the analytical techniques. One NS was removed because of a failure in sample preparation.

### Study subjects

The study population consisted of 16 healthy non-smokers with no smoking history (NS), 17 current tobacco cigarette smokers (≥ 10 pack-years) without respiratory symptoms or airflow limitation (S), and 17 subjects with respiratory symptoms and airflow limitation (COPD) (Table 1). COPD was diagnosed according to the criteria recommended by the National Institutes of Health (NIH). Global Initiative for Chronic Obstructive Lung Disease (GOLD) stages II (*n* = 6), III (*n* = 8), and IV (*n* = 3) were included. Exclusion criteria were the presence of other lung diseases and other systemic inflammatory diseases than COPD and acute infections or exacerbations within the last two months before the blood samples were taken. Exclusion criteria were also the use of systemic steroids or immunosuppressive drugs in the last 4 weeks before sample preparation. Age was > 40 years in all groups. The study was approved by the ethics committee of the Ruhr-University Bochum, Germany (4257-12, 4772-13) and all subjects gave their written consent.

**Table 1:** Demographics of study subjects

Parameter	NS	S	COPD
N	16	17	17
age [yrs]	58.7 ± 14.1	54.9 ± 8.4	62.8 ± 7.9
sex [m: f]	6:10	9:8	8:9
FEV1 [% pred.]	108.6 ± 16.3	102.3 ± 23.4	50.1 ± 19.6^****§§§§^
FVC [% pred.]	108.2 ± 13.6	102.8 ± 14.9	78.1 ± 18.2^***§§^
FEV1/FVC [%]	81.2 ± 7.6	79.3 ± 5.8	52.4 ± 12.9^****§§§§^
pack years	0	35.7 ± 18.2	46.8 ± 22.6
CS: FS	-	17:0	6:11
monocytes [% WBC]	4.3 ± 2.5	3.8 ± 1.5	4.9 ± 1.9
lymphocytes [% WBC]	30.6 ± 10.0	33.2 ± 11.9	28.0 ± 10.2
neutrophils [% WBC]	63.3 ± 11.2	63.0 ± 13.9	64.9 ± 10.0
Eosinophils [% WBC]	1.9 ± 1.8	1.8 ± 1.3	2.0 ± 1.7

*COPD* Chronic obstructive pulmonary disease, *NS* Healthy never-smokers, *S* Current tobacco cigarette smokers without obstructive lung disease (≥ 10 pack-years), *FEV1* Forced expiratory volume 1, *FVC* Forced vital capacity, *CS* Current tobacco cigarette smokers, *FS* Former tobacco cigarette smokers, *WBC* White blood cells. Data are given as mean ± SD. FEV1 [% pred.], FVC [% pred.], FEV1/FVC [%]: *One way* ANOVA each *P* < 0.0001, post hoc: *Dunn´s multiple comparison test*, COPD vs. NS: ***P < 0.001, ****P < 0.0001; COPD vs. S: ^§§^*P* < 0.01, ^§§§§^*P* < 0.0001

### Isolation and in vitro infection of PBMCs

PBMCs were isolated via Ficoll-based gradient centrifugation as described (Knobloch et al., 2011). Briefly, 80 ml whole acid citrate dextrose-blood was taken from the peripheral veins of subjects and was diluted 1:1 in 0.9% NaCl solution. 40 ml of this were then placed on top of a 5 ml Ficoll layer. The cells were then centrifuged at 1700 rcf without brake for 60 min. The PBMC fraction was removed from the gradient using a plastic pipette. The cells were washed with PBS and the pellet was resuspended in 5 ml ACK lysis buffer (Lonza) to remove remaining erythrocytes. After 10 min, 20 ml PBS was added and the cells were centrifuged for 20 min at 1700 rcf at 4 °C. After another washing step, the cell pellet was then resuspended in 5 ml RPMI media to count the cells and to check the vitality (> 85%). They were seeded in 48 well plates at 5 × 10^5^ cells per well in 500 µl RPMI medium without FCS. Then, the cells were infected with RSV (Virapure, San Diego, CA, USA, Lot: C1511B) at MOI 0.1 or 1.0 (input). To this end, RSV was diluted in serum-free RPMI and was added to the cells directly after seeding at 37° C without shaking. Uninfected cells were used as a control. 16 h post infection, 10% FCS was added to the wells. Samples were harvested after 24 h and after 7 days. Cells were scraped off on ice and transferred to an Eppendorf tube together with the supernatant. After centrifugation for 5 min at 5000 rcf and 4 °C, the supernatant was transferred to another tube and used for cytokine analyses. 50 µl of the supernatant was used for RNA isolation to measure viral replication. RNA was also isolated from the cell pellet to analyze ISG expression. All samples were stored at −80 °C until analysis.

### RNA isolation and cDNA synthesis

RNA isolation from cell pellets and supernatants were performed with the RNeasy Mini Kit (Qiagen, Hilden Germany) or with the NucleoSpin RNA Virus KIT (Machery Nagel) respectively, according to manufacturer’s instructions with the use of DNaseI. RNA concentrations were determined using a NanoDrop1000 (Nanodrop) according to the manufacturer’s instructions. Samples were stored at −80 °C. cDNA synthesis was performed with an Omniscript RT KIT (Qiagen) according to the manufacturer’s instructions. The cDNA was stored at −80 °C.

### Taqman real time PCR

To analyse ISG gene expression in reference to the 18 S RNA housekeeping gene, taqman real-time PCR (7500 Fast Real-Time PCR System, Applied Biosystems) was used. The qPCR was performed on MicroAMp Fast 96-well plates (Applied Bioscience) using the Quantitect Probe PCR Kit (Qiagen) according to the manufacturer instructions. Primer sequences are given in Table 2.

**Table 2:** Primer sequences for Taqman real time PCR

Gene	Forward primer (5´−3´)	Reverse primer (5´−3´)	Probe (5′-FAM, 3′-TAMRA)
18 S rRNA	CGCCGCTAGAGGTGAAATTCT	CATTCTTGGCAAATGCTTTCG	ACCGGCGCAAGACGGACCAGA
Viperin	CACAAAGAAGTGTCCTGCTTGGT	AAGCGCATATATTTCATCCAGAATAAG	CCTGAATCTAACCAGAAGATGAAAGACTCC
MXA	CAGCACCTGATGGCCTATCAC	CATGAACTGGATGATCAAAGG	AGGCCAGCAAGCGCATCTCCAG
OAS	CTGACFCTGACCTGGTTGTCT	CCCCGGCGATTTAACTGAT	CCTCAGTCCTCTCACCACTTTTCA

### Rotorgene reverse transcription quantitative PCR (RT-qPCR)

For the quantification of RSV in supernatants, the RNA was isolated at days 1 and 7 and subjected to RT-qPCR with the QuantiTect KIT (Qiagen) according to the instructions of the manufacturer with SYBR^®^Green. Primer sequences were 5´- AGATCAACTTCTGTCATCCAGCAA − 3´; 5- GCACATCATAATTAGGAGTATCAAT-3´. The protocol has been published before (Ternette et al. 2007).

### ELISA

Cytokine concentrations in supernatants were measured by ELISA. For CCL2, IFNα, IFNγ, IL-1β, TNFα, IL-6, IL-8, and GM-CSF Ready-Set-Go KITs from eBioscience were used. For CCL5, the DuoSet KIT from R&D Systems was used.

### Statistical analyses

Statistical analyses were performed using the Graph Pad Prism 7 software. To calculate the normal distribution of the data, D’Agostino & Pearson normality test was performed. The tests used are given in the legends of the tables or figures.

## Results

### Cytokine baseline levels were increased in PBMCs of COPD subjects

PBMCs were cultured for 24 h and 7 days. In the following, we describe the statistically significant differences in cytokine production between the groups. Without further treatment, IFNα and IL-1β were increased in COPD compared to NS and S at both times (Table 3). IL-6 was increased in COPD compared to NS and S after 24 h and compared to S after 7 days (Table 3). IFNγ was increased in COPD compared to NS after 24 h and without differences between the groups after 7 days. TNFα, however, was reduced in COPD and in S compared to NS (Table 3). CCL2, CCL5, and IL-8 were without differences between the groups at both times (Table 3). These data indicate that PBMCs contribute to systemic inflammation in COPD.

**Table 3:** Baseline cytokine levels in cultured PBMCs

Cytokine	Time	concentration [pg/ml]; median, interquartile range					
		NS		S		COPD	
CCL2	24 h	2886	1128; 3827	2536	1693; 3943	2643	1635; 3283
	7 d	13,737	6283; 20,279	31,943	5931; 73,035	6390	3403; 17,564
CCL5	24 h	1761	1036; 2628	1608	867.2; 2859	1192	907.4; 1448
	7 d	168.5	78.64; 230.3	176	149.6; 241.1	132,2	94.23; 184.8
IFNα	24 h	**15.32**	**11.64; 20.92**	**13.86**	**10.61; 24.99**	**27.15**	22.07; 34.70*** ^**§§**^
	7 d	**14.00**	**10.73; 18.76**	**11.08**	**8.31; 24.71**	**22.72**	20.09; 24.63*** ^**§§**^
IFNγ	24 h	**119.1**	**30.14; 238.0**	**203.4**	**95.75; 282.5**	**237.4**	219.0; 336.0**
	7 d	78.03	43.61; 308.2	230.1	128.8; 482.7	225.6	185.4; 284.9
IL-1β	24 h	**33.68**	**24.75; 44.73**	**24.54**	**9.75; 44.64**	**62.54**	35.08; 146.3* ^**§§**^
	7 d	**27.73**	**23.11; 39.85**	**13.69**	**9.52; 37.12**	**50.15**	31.43; 81.37* ^**§§§**^
IL-6	24 h	**615.6**	**236.7; 1176**	**289.8**	**184.6; 524.2**	**2605**	909.2; 4404* ^**§§§§**^
	7 d	**649.9**	**208.0; 1484**	**247.1**	**196.5; 559.6**	**1739**	**737.9; 3973** ^**§§§§**^
IL-8	24 h	48,700	30,001; 1.06E5	51,102	20,771; 76,141	70,278	34,259; 1.75E5
	7 d	1.10E5	63,633; 3.03E5	1.10E5	47,524; 2.20E5	1.15E5	48,299; 3.56E5
TNFα	24 h	**297.9**	**257.7; 661.6**	**224.0**	**76.00; 324.9** ^**#**^	**220.4**	173.3; 263.6*
	7 d	**232.8**	**166.0; 291.3**	**130.5**	**99.21; 188.7** ^**##**^	**159.0**	123.9; 190.6*

Cells were cultured for the indicated time. Cytokine concentrations were measured in supernatants by ELISA. Data were compared between the groups with Kruskal-Wallis test (bold, *p* < 0.05) and post hoc Benjamini Hochberg correction. COPD vs. NS: *p < 0.05, **p < 0.01, ***p < 0.001; COPD vs. S: ^§^*p* < 0.05, ^§§^*p* < 0.01, ^§§§^*p* < 0.001, ^§§§§^*p* < 0.0001; S vs. NS: ^#^*p* < 0.05, ^##^*p* < 0.01; *NS* Never smoker, *S* Current tobacco cigarette smoker, *COPD* Chronic obstructive pulmonary disease

### RSV induced cytokines of the innate and of the Th1 immune response

The response of the cultured PBMCs to in vitro infection with RSV at MOI 0.1 and 1.0 was analyzed after 24 h and seven days. The concentrations of CCL2, CCL5, IFNα, IFNγ, IL1-β, IL-6, IL-8, and TNFα were increased in the presence of RSV in the culture supernatants of NS, S, and/or COPD compared to untreated cells (supplementary Table 1). In order to analyze for the effect of RSV we subtracted the baseline data. RSV induced all of the above-mentioned innate immune and Th1-related cytokines in a concentration and time dependent manner in NS (Table 4). The Th2 markers IL-4 and IL-5, the Th17 marker IL-17, as well as GM-CSF and TGFβ were below the detection limit (data not shown) and, thus, might not be induced by RSV. Therefore, these cytokines were not considered further.

**Table 4:** RSV-induced cytokine secretion in PBMCs

cytokine	RSVMOI	Time	△ concentration (induction minus baseline); median, interquartile range					
			NS		S		COPD	
CCL2 [ng/ml]	0.1	24 h	0.448	0.135; 2.951^††^	0.187	−0.061; 1.217	1.803	0.215; 9.277^††^
		7 d	46.68	13.21; 92.01^††††^	22.44	3.118; 59.73^†††^	27.87	1.511; 103.4^††^
	1.0	24 h	1.171	−0.272; 2.641^†^	1.233	0.129; 2.868^†^	1.909	0.003; 6.124^††^
		7 d	46.04	−0.845; 188.9^††^	64.16	24.58; 100.7^††††^	5.615	−3.438; 50.99
CCL5 [pg/ml]	0.1	24 h	**140.7**	** −25.80; 623.3**	**21.95**	** −318.3; 80.30**	**730.8**	**155.1; 1091** ^**††† §§§**^
		7 d	**35.72**	** −15.07; 279.3**	**19.93**	** −14.87; 124.2**	**800.3**	**49.40; 1325** ^**† §**^
	1.0	24 h	**243.3**	** −43.67; 864.7**	**15.14**	** −59.83; 278.8**	**1125**	**834.7; 1740** ^**††††**^ * ^**§§§**^
		7 d	**98.34**	** −7.745; 650.3** ^**††**^	**41.79**	**1.870; 140.4** ^**†**^	**1688**	**194.3; 3712** ^**††† §§**^
IFNα [pg/ml]	0.1	24 h	** −0.145 **	** −1.485; 95.55**	** −1.340 **	** −3.465; 0.140**	**27.13**	**11.73; 91.78** ^**††††**^ * ^**§§§**^
		7 d	**2.795**	**−1.025; 25.89** ^**†**^	**1.190**	** −0.575; 2.160**	**32.07**	**7.630; 103.9** ^**††††**^ * ^**§§**^
	1.0	24 h	**1.565**	** −1.343; 1078**	** −0.550 **	** −2.115; 11.79**	**436.3**	**18.27; 619.2** ^**††††**^ ** ^**§§§**^
		7 d	**2.425**	**0.850; 862.1** ^**†**^	**2.350**	** −2.300; 6.610**	**331.8**	**19.76; 525.7** ^**†††**^ * ^**§§**^
IFNγ [pg/ml]	0.1	24 h	**7.350**	** −24.62; 46.45**	** −5.920 **	** −35.32; 3.970**	**119.1**	**9.460; 422.1** ^**†† §§**^
		7 d	238.1	6.705; 6560^††^	239.8	114.6; 891.6^††††^	1252	208.0; 3255^††††^
	1.0	24 h	**11.39**	** −10.74; 3595**	** −5.190 **	** −39.47; 11.91**	**525.3**	**19.67; 1827** ^**†† §**^
		7 d	502.7	25.62; 3471^†††^	650.2	385.4; 3569^††††^	848.8	122.4; 1541^††††^
IL-1β [pg/ml]	0.1	24 h	**1.485**	** −3.143; 455.7**	**0.290**	** −3.670; 2.520**	**290.6**	**4.100; 958.2** ^**††† §§**^
		7 d	**3.335**	**1.413; 98.11** ^**††**^	**1.430**	** −0.175; 5.615**	**230.2**	**4.230; 763.5** ^**†† §**^
	1.0	24 h	**3.245**	** −2.588; 2429**	**0.940**	** −3.315; 3.695**	**1883**	**7.200; 4939** ^**††† §§**^
		7 d	**1.595**	** −0.280; 1963**	** −0.690 **	** −4.720; 2.270**	**1747**	**10.20; 4417** ^**††† §§**^
IL-6 [ng/ml]	0.1	24 h	**0.121**	** −0.0265; 3.942**	**0.007**	** −0.084; 0.048**	**5.770**	**0.275; 40.39** ^**†††**^ * ^**§§§§**^
		7 d	**0.358**	**0.010; 3.005** ^**††**^	**0.070**	**0.037; 0.162** ^**†**^	**12.91**	**0.445; 69.26** ^**†††**^ * ^**§§§**^
	1.0	24 h	**0.290**	**0.0855; 5.097** ^**†**^	**0.050**	** −0.007; 0.287**	**8.386**	**0.625; 33.70** ^**†††**^ * ^**§§§§**^
		7 d	**0.279**	**0.092; 11.55** ^**†††**^	**0.191**	** −0.018; 0.448**	**13.39**	**0.379; 57.18** ^**††††**^ * ^**§§§**^
IL-8 [ng/ml]	0.1	24 h	**4.160**	** −3.373; 23.02**	**1.256**	** −5.070; 10.62**	**82.39**	**9.757; 346.1** ^**†††**^ * ^**§§**^
		7 d	**115.0**	**29.68; 741.4** ^**††**^	**28.21**	** −5.047; 84.62** ^**#**^	**464.6**	**12.08; 1023** ^**†††**^
	1.0	24 h	**1.567**	** −12.32; 14.10**	**6.116**	** −19.12; 12.51**	**36.68**	** −5.891; 98.34** ^**† §§**^
		7 d	78.47	7.893; 583.1^††^	13.80	−8.379; 102.5	91.51	0.000; 386.0^††^
TNFα [ng/ml]	0.1	24 h	**0.224**	**0.026; 0.975** ^**††**^	** −0.015 **	** −0.103; 0.023** ^**#**^	**1.764**	**0.098; 4.521** ^**††† §§§§**^
		7 d	0.064	−0.018; 0.071	0.006	−0.033; 0.051	−0.003	−0.012; 0.067
	1.0	24 h	**0.180**	**−0.046; 6.583** ^**†**^	**−0.038**	**−0.124; 0.029** ^**#**^	**9.374**	**0.289; 13.01** ^**††† §§§§**^
		7 d	**0.007**	** −0.031; 0.321**	** −0.029 **	**−0.088; 0.005** ^**† #**^	**0.111**	**0.000; 0.312** ^**†† §§§**^

Cells were cultured for the indicated time in the presence of RSV at the indicated multiplicities of infections (MOIs). Cytokine concentrations were measured in supernatants by ELISA. Baseline levels (Table 3) were subtracted. Data were compared to a hypothetical value of 0 (= no difference to baseline) with Wilcoxon signed rank tests: ^†^*p* < 0.05, ^††^*p* < 0.01, ^†††^*p* < 0.001, ^††††^*p* < 0.0001. Data were compared between the groups with Kruskal-Wallis test (bold, *p* < 0.05) and post hoc Benjamini Hochberg correction. COPD vs. NS: *p < 0.05, **p < 0.01; COPD vs. S: ^§^*p* < 0.05, ^§§^*p* < 0.01, ^§§§^*p* < 0.001, ^§§§§^*p* < 0.0001; S vs. NS: ^#^*p* < 0.05. NS, healthy never smoker; S, current tobacco cigarette smoker; COPD, chronic obstructive pulmonary disease

### RSV-induced CCL2 was not significantly different between NS, S and COPD

To compare the responses to RSV between the groups we used the baseline-subtracted data. CCL2 was concentration-dependently induced by RSV at both time points. This effect was observed in all groups without statistically significant differences (Table 4).

### RSV induced CCL5 and IFNγ earlier and stronger in COPD than in NS and/or S

RSV induced CCL5 and the type 1 cytokine IFNγ after 24 h in COPD but not in NS, and S (Table 4). If compared between the groups, baseline-subtracted CCL5 was higher in COPD compared to NS and S, and baseline-subtracted IFNγ was higher in COPD compared to S (Table 4). After seven days, RSV induced CCL5 and IFNγ in all groups (Table 4). For CCL5 this effect was stronger in COPD compared to S, and for IFNγ this was without differences between the groups (Table 4).

### RSV induced IFNα and IL-8 earlier and stronger in COPD than in NS but not in S

RSV induced the anti-viral cytokine IFNα and the neutrophil activator IL-8 after 24 h in COPD but not in NS, and S (Table 4). Baseline-subtracted IFNα and IL-8 were higher in COPD compared to NS, and S (Table 4). After seven days, RSV induced IFNα and IL-8 in NS and in COPD but not in S (Table 4). The effects on IFNα were stronger in COPD compared to NS, and S (Table 4). The effects on IL-8 were stronger in NS compared to S (Table 4).

### RSV induced IL-1β earlier in COPD than in NS but not in S

RSV induced IL1β after 24 h in COPD but not in NS, and S (Table 4). Baseline-subtracted IL-1β was higher in COPD compared to S (Table 4). After seven days, RSV induced IL-1β in NS and in COPD but not in S (Table 4). The effects on IL-1β were stronger in COPD compared to S (Table 4).

### RSV induced IL-6 stronger in COPD compared to NS and S

After 24 h, RSV induced IL-6 in NS and in COPD but not in S (Table 4). After seven days, RSV induced IL-6 concentration-dependently in all three groups (Table 4). The effects were stronger in COPD compared to NS and S at all conditions (Table 4).

### RSV induced TNFα longer in COPD than in NS but not in S

After 24 h, RSV induced TNFα in NS and in COPD but not in S (Table 4). After seven days, TNFα was induced concentration-dependently by RSV in COPD, reduced in S, and remained unchanged in NS (Table 4). The differences in the effects of RSV between the groups were statistically significant.

### RSV replication in PBMCs was increased in COPD and reduced in S

**Fig. 1:**
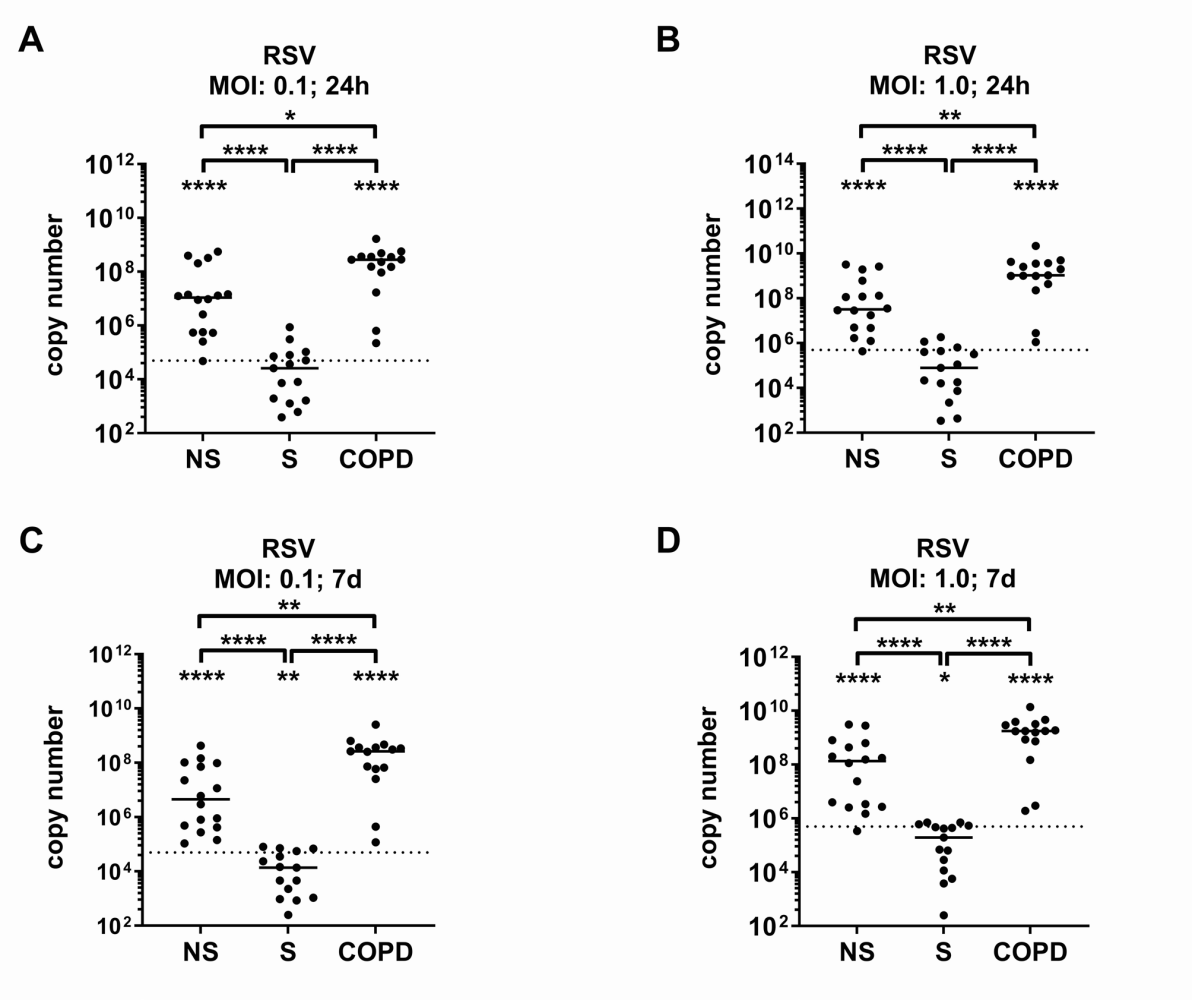
RSV replication in PBMCs. PBMCs from healthy never smokers (NS), current tobacco cigarette smokers without airflow limitation (S), and COPD subjects (5 × 10^5^cells per approach) were infected with RSV-A2 at the given multiplicities if infection (MOI). After 24 h and 7 days replication was measured by real time RT-RCR. Data are presented as median with scatter. N numbers are according to Table 1, two probes of S and two probes of COPD were excluded because of technical errors or insufficient sample material. Data were analyzed with Wilcoxon signed rank tests (vs. input) and successive Mann Whitney tests (order: NS vs. COPD, NS vs. S, S vs. COPD). *P < 0.05; **P < 0.01; ***P < 0.001; ****P < 0.0001 compared to input (dashed line) if placed on top of bars or between groups as indicated. Dashed line: RSV input

Next, we investigated RSV copy numbers. After 24 h of infection, the copy numbers in PBMCs were increased in NS and COPD but not in S compared to the RSV input (Fig. 1A, B). After 7 days, the copy numbers were increased in NS and COPD but reduced in S compared to input (Fig. 1C, D). At all conditions, the copy numbers were higher in COPD compared to NS and S, and were higher in NS compared to S. (Fig. 1). This suggests RSV replicates in PBMCs and might do more efficient in COPD but less efficient in smokers without COPD, which might cause the differences in the cytokine response between the groups.

### The expression of ISGs is changed in COPD

**Fig. 2:**
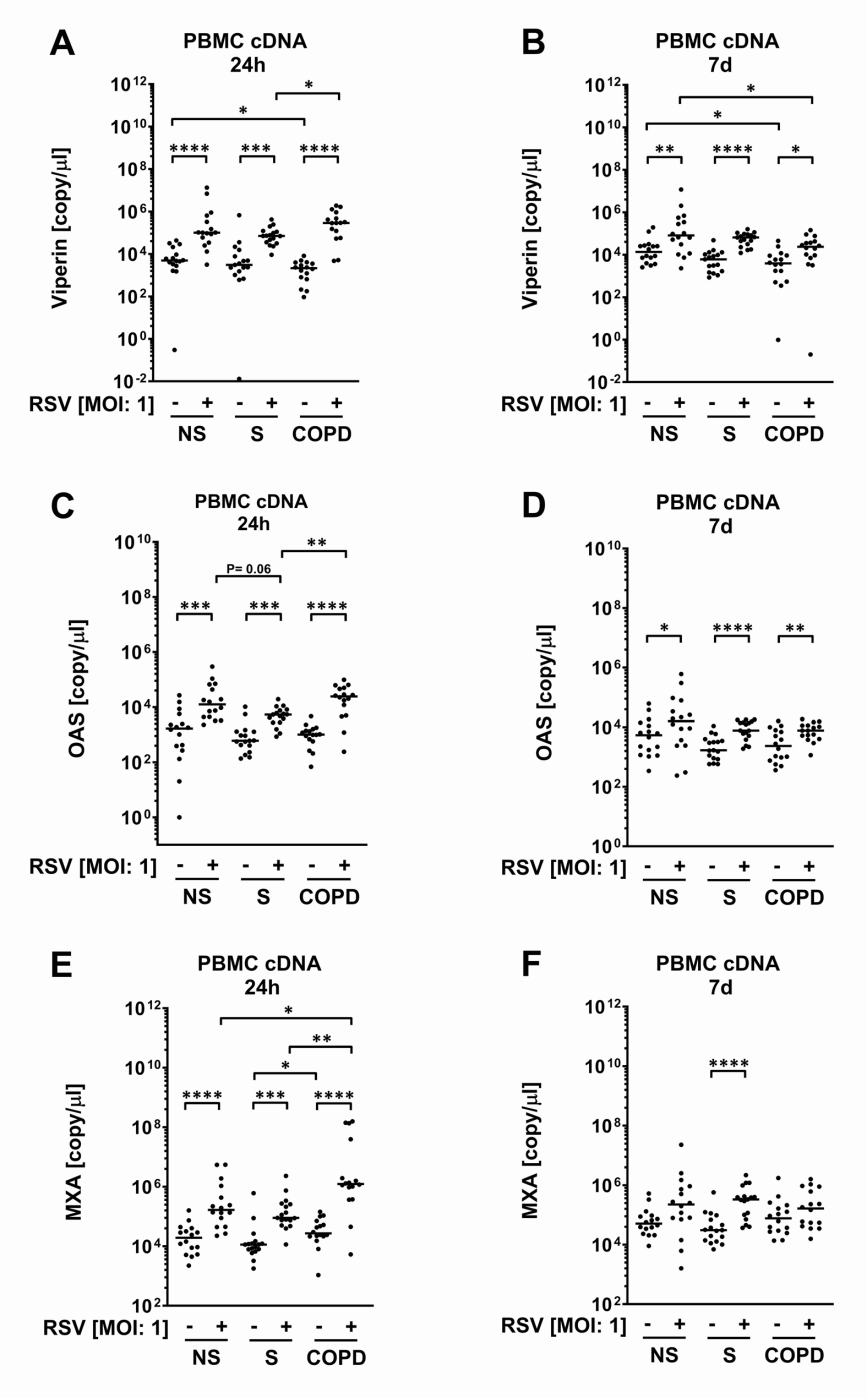
ISG gene expression in response to RSV in PBMCs. PBMCs from never smokers (NS), current tobacco cigarette smokers without airflow limitation (S), and COPD subjects were infected with RSV-A2 at a multiplicity of infection (MOI) of 1. After 24 h and 7 days, the mRNA levels of Viperin (**A**, **B**), OAS (**C**, **D**), and MXA (**E**, **F**) were measured by Taqman real-time RT-PCR. Data are presented as median with scatter. N numbers are according to Table 1, one probe of COPD was excluded because of insufficient sample material. The data were compared by Wilcoxon T-test and by Kruskal-Wallis test with post hoc Dunn test. *P < 0.05; **P < 0.01; ***P < 0.001; ****P < 0.0001

Next, we investigated the expression of three ISGs. Compared to NS, baseline viperin mRNA levels after 24 h and after seven days were both reduced in COPD (Fig. 2A, B). RSV induced viperin mRNA in all groups (Fig. 2A, B). After 24 h, this effect was increased in COPD compared to S but without differences compared to NS (Fig. 2A). After seven days of RSV infection, viperin mRNA was reduced in COPD compared to NS (Fig. 2B). Baseline OAS was without differences between the groups (Fig. 2C, D). RSV induced OAS in all groups (Fig. 2C, D). This effect was reduced by trend or significantly in S compared to NS or COPD, respectively, after 24 h (Fig. 2C). RSV-induced OAS was without differences between the groups after seven days (Fig. 2D). Baseline MxA after 24 h was lower in S compared to COPD but without difference to NS (Fig. 2E). There were no differences between the groups after seven days (Fig. 2F). RSV induced MxA in all groups after 24 h but significantly only in S after seven days (Fig. 2E, F). The effect after 24 h was increased in COPD compared to NS, and S (Fig. 2E).

## Discussion

PBMC cytokine baseline levels might not significantly contribute to local infection defense processes because it requires signals from the local inflammation for the recruitment of circulating immune cells to the sites of infection that can influence their cytokine gene expression. However, higher baseline levels might contribute to the systemic inflammation that is present in COPD. Systemic inflammation in COPD is particularly characterized by increased TNFα, IL-1β, IL-6, IL-8, and IFNγ serum levels (MacNee 2013; Moermans et al. 2011; Sinden et al., 2010; Zou et al. 2017). It is a matter of discussion whether this is due to an overspill of the massively increased cytokine levels in the lungs, to increased immune cell numbers in the blood of COPD subjects or to increased production levels of one or several circulating cell types (MacNee 2013; Moermans et al. 2011; Sinden et al., 2010; Zou et al. 2017). A major role in this context has been attributed to circulating neutrophils (MacNee 2013; Moermans et al. 2011; Sinden et al., 2010; Zou et al. 2017), which, however, were not part of the PBMC fractions used in our study. Our data indicate that increased baseline levels of IL-1β, IL-6 and IFNγ of cells of the PBMC fraction might contribute to systemic inflammation. IL-1β and IL-6 might be produced by monocytes or dendritic cells whereas IFNγ could result from T-cells or natural killer cells. IL-8 and TNFα baseline levels were not up-regulated in PBMCs from COPD subjects. Increased circulating IL-8 and TNFα in COPD might therefore come from neutrophils that are not part of the PBMC fraction but are known to produce these COPD key factors (Kaiser et al. 2021). Serum data for IFNα in COPD are, to our knowledge, not yet available, and it is also unclear if this innate immune response cytokine contributes to systemic inflammation. Therefore, the relevance of our observation of increased IFNα baseline levels from PBMCs of COPD subjects is unclear.

The IFNα, Th1 and inflammatory responses to RSV are central for defense and have at least partially been pictured in our PBMC culture model. RSV induced IFNα, the key Th1 factor IFNγ and several inflammatory cytokines suggesting that our cell culture model is suitable for investigating immune pathologies in infection defense mechanisms to RSV in COPD. However, neither the Th17 part that is associated with Tgfβ-dependent remodeling processes (Chakir et al. 2003) nor the deleterious Th2 response was observed in this experimental model. This is a limitation of the model on the one hand but allows on the other hand to focus on the important IFNα- and Th1-dependent processes of the RSV immune response. We hypothesized that the high inflammatory state of circulating immune cells in COPD might impair their response to RSV. Our data confirmed the hypothesis and showed that RSV infection further increases the inflammatory state, because the cytokine response overall is stronger and earlier in COPD compared to the cells of healthy subjects. Besides IFNα, particularly the inflammatory cytokine response in terms of IL-6 and IL-8 is stronger in PBMCs of COPD subjects. Both cytokines are key factors of the local inflammation in stable and exacerbated COPD (Barnes 2009). Given that our experimental model of in vitro RSV infection of PBMCs reflects responses after recruitment to the sites of infection, we might have found a cytokine overproduction in immune cells that contributes to the increase in inflammation in RSV-caused exacerbations in COPD.

IFNy and CCL5 are both expressed by Th1 and Tc1 cells as well as by subsets of innate lymphocytes (ILCs) (Mariani et al. 2002). Considering the time that is required for naive T-cells to get activated, the responses after 24 h might rather come from the innate lymphocytes or from a re-activation of RSV-specific memory T-cells. Thus, our data showing IFNγ and CCL5 release after RSV infection for 24 h in COPD but not in S and NS indicate for an overactivation of innate lymphocytes and/or specific memory T-cells in response to RSV in COPD. After seven days of infection, there are no statistically significant differences between COPD and NS suggesting that the IFNγ and CCL5 response of lymphocytes in the PBMCs might not be different and overwrite the effects seen after 24 h. Both, IFNγ and CCL5 might contribute to COPD local inflammation in stable and exacerbated stages (Barnes 2009; Costa et a., 2008). Thus, our data provide indication that lymphocytes, ILCs and/or RSV-specific memory T-cells contribute to RSV exacerbations by releasing more IFNγ and CCL5 than it is required for effectively combating RSV.

In PBMCs, IL1β is mainly produced by monocytes (Cooper et al. 2001), and data from mouse models suggest that it might contribute to airway inflammation and remodeling processes in the COPD lung (Lappalainen et al. 2005). Our data suggest that IL-1β production and release in response to RSV infection starts earlier in recruited circulating cells in COPD compared to healthy subjects indicating a pathologically rapid activation of monocytes or other cells capable of IL-1β production that might contribute to the increase in inflammation in RSV-induced exacerbations. This rapid IL-1β activation in COPD might explain the likewise early IFNγ production we have shown, because IL-1β is known as a co-inducer of IFNγ production in NK cells (Cooper et al. 2001). Therefore, we carefully speculate that our data indicate for an overactivation of the monocyte-NK cell axis in RSV-induced COPD exacerbations.

TNFα is a central player in airway inflammation in stable and exacerbated COPD (Cazzola et al. 2021). Our data indicate an abnormally longer TNFα production from mononuclear immune cells in response to RSV infection in COPD. Concerning the other cytokines that we have investigated there is no indication in COPD for a prolonged activation of either monocytes or Th1 cells, the two major TNFα producers in the PBMC fraction (Cazzola et al. 2021), after RSV infection. Thus, the source of the longer TNFα production remains unclear. Nonetheless, this molecular pathology might contribute to the increase in local TNFα levels in associated COPD exacerbations.

Surprisingly, the overall cytokine response was lowest in active tobacco cigarette smokers without COPD. This matches the replication data that showed RSV replication in cells of COPD and NS but not in cells of active smokers without airflow limitation. RSV replication was, as cytokine release, increased in COPD compared to NS. We conclude that the efficacy of RSV replication significantly determines the amount of cytokine production. In epithelial cells, cigarette smoking enhances RSV replication making it to an important risk factor for RSV-induced airway disease and exacerbations (Groskreutz et al. 2009). This is contrary to our findings in PBMCs. Indeed, ICAM-1, an RSV entry receptor, has shown to be increased by active smoking in PBMCs but decreases after smoking cessation (Yeh et al. 2021), and the expression of CX3 CL1, another RSV entry receptor, is also induced by smoking (Zhang et al., 2010). Subgrouping of our COPD group in current and former smokers did not show significant differences in replication in PBMCs (data not shown). Therefore, the molecular reason for the increase in replication in PBMCs of COPD subjects and its missing in current smokers without COPD remains to be investigated.

According to previous studies about the regulation and downstream effects of ISGs (Schoggins 2019), we speculated that rapid upregulation of IFNα in response to RSV might induce ISG expression in PBMCs, which then suppresses replication. The resulting viral load might determine the amount of inflammatory cytokine production. The expression of all investigated ISGs was induced in all three groups in response to infection after 24 h and, with exception of MxA in NS and COPD, also after 7 days. The inductions after 24 h in NS and S were surprising because we did not observe an IFNα upregulation early at this time but only later after seven days in these groups. Also, IFNγ, which also might also contribute to the induction of ISG expression (Costa-Pereira et al. 2002), was not upregulated in NS, and S after 24 h. We conclude that other cytokines like IFNβ might be largely responsible for early ISG gene expression in PBMCs.

RSV-induced MxA was increased in COPD after 24 h, which could be explained by the IFNα overproduction in this group. In contrast, viperin and OAS, both might not respond to the pathological IFNα upregulation according to our data. However, we cannot exclude that a putative response occurred outside the chosen time points of analyses. Our observation that despite the increased MxA levels RSV replication is increased in COPD supports the data indicating that the replication of RSV might be resistant to the blocking effects of MxA (Atreya et al., 1999). We did not observe an overexpression of the three tested ISG genes in S that could explain the lack of replication in this group. Therefore, either other ISGs might be specifically up-regulated in active smokers or the reason is independent from ISGs, which might be more likely because we also did not observe an overproduction of IFNα, the major ISG regulator, in S. Baseline viperin levels were reduced in COPD compared to NS as where RSV-induced viperin expression after seven days. This low amount of viperin could help to explain the increased RSV replication in COPD. OAS is not differentially regulated in COPD compared to NS and therefore might not influence the increased replication in COPD. Systemic is different to local ISG expression because in sputum probes viperin is increased, OAS is reduced and MxA is without differences in COPD compared to healthy subjects (Hilzendeger et al. 2016). This indicates that immune cells freshly recruited after infection could have a different pathological ISG profile than local immune cells in COPD.

## Conclusion

In summary, we have found that circulating immune cells in COPD have an increased baseline cytokine production and an increased cytokine response to RSV. The latter might be explained by differences in the efficacy of RSV replication in PBMCs and in part by differences in ISG expression. Given that circulating immune cells in vivo interact with RSV after their recruitment to the site of infection, we might have found a molecular pathology that contributes to the increase in inflammation in exacerbations. Therefore, our study provides indication for drug targets in circulating cells in this context.

## Supplementary Information

Supplementary Material 1.

## Data Availability

The original data and the analyses of the data that are presented in this study are available from the corresponding author on request.

## Abbreviations

CCL2/5: CC-chemokine ligand 2/5
COPD: Chronic Obstructive Pulmonary Disease
CS: Current tobacco cigarette smokers
ELISA: Enzyme-linked Immunosorbent Assay
FCS: Fetal calf serum
FEV1: Forced expiratory volume in 1 s
FS: Former tobacco cigarette smokers
GM-CSF: Granulocyte Macrophage Colony Stimulating Factor
FVC: Forced vital capacity
GOLD: Global Initiative for Chronic Obstructive Lung Disease
ICAM-1: Intercellular adhesion molecule 1
IFNα/β/γ: Interferon α/β/γ
IL-1β/-4/-5/-6/-8/-17: Interleukin 1β/-4/-5/-6/-8/-17
ILCs: Innate lymphocytes
ISG: Interferon-stimulating genes
MOI: Multiplicity of infection
MxA: Myxovirus resistant gene A
NIH: National Institutes of Health
NK cells: Natural killer cells
NS: Healthy never smokers
OAS: 2’,5’-oligoadenylate synthase
PBMCs: Peripheral blood mononuclear cells
PCR: Polymerase chain reaction
pred.: Predicted
RSV: Respiratory Syncytial Virus
RT-PCR: Reverse transcriptase polymerase chain reaction
RT-qPCR: Rotorgene reverse transcription quantitative PCR
S: Current smokers without airway obstruction
Tc1: Cytotoxic T cell type 1
Th1/2: T helper cell type 1/2
TGFβ: Transforming growth factor β
TNFα: Tumor necrosis factorα
WBC: White blood cells
