# Supplementary material for: Cytokine production of mononuclear leukocytes in response to respiratory syncytial virus is increased in COPD but suppressed in non-COPD tobacco smokers
Source: Mol Med. 2025 Jun 12;31:237. doi: 10.1186/s10020-025-01277-4 (PMC12160369; doi:10.1186/s10020-025-01277-4)
Supplement: Supplementary file 1 — Supplementary Material 1. [file 10020_2025_1277_MOESM1_ESM.pdf]

Supplementary Information

Supplementary table 1: Cytokine levels in cultured PBMCs infected with RSV

**cytokine**	**RSV MOI**	**time**	**concentration [ng/ml]; median, interquartile range**					
			**NS**		**S**		**COPD**	
CCL2	0.1	24 h	2.969	1.594; 6.758††	2.998	1.834; 4.724	4.656	2.324; 11.53††
		7 d	77.71	26.58; 107.4††††	73.01	25.74; 127.3†††	69.17	10.35; 119.4††
	1.0	24 h	3.651	3.047; 5.538†	3.349	2.736; 7.372†	3.639	3.020; 9.345††
		7 d	**52.23**	**13.18; 209.4††**	**128.3**	**49.88; 160.9††††**	**15.82**	**3.711; 64.30§**
CCL5	0.1	24 h	1.438	1.111; 2.635	1.518	0.908; 2.272	1.972	1.101; 2.635†††
		7 d	**0.216**	**0.164; 0.380**	**0.211**	**0.155; 0.360**	**0.935**	**0.331; 1.461††† * §§**
	1.0	24 h	1.584	1.211; 3.454	1.661	1.207; 2.531	2.588	1.818; 3.330††††
		7 d	**0.287**	**0.160; 0.702††**	**0.219**	**0.154; 0.327†**	**1.747**	**0.577; 3.865†††† * §§§**
IFNα	0.1	24 h	**0.017**	**0.014; 0.116**	**0.012**	**0.010; 0.250**	**0.054**	**0.042; 0.115†††† ** §§§§**
		7 d	**0.015**	**0.010; 0.045†**	**0.012**	**0.009; 0.029**	**0.054**	**0.036; 0.123††† ** §§§§**
	1.0	24 h	**0.018**	**0.012; 1.095**	**0.019**	**0.012; 0.036**	**0.455**	**0.061; 0.644†††† ** §§§§**
		7 d	**0.014**	**0.012; 0.088†**	**0.016**	**0.011; 0.026**	**0.354**	**0.050; 0.548††† ** §§§**
IFNγ	0.1	24 h	**0.111**	**0.036; 0.232**	**0.211**	**0.076; 0.304**	**0.346**	**0.238; 0.754†† ** §§**
		7 d	0.319	0.085; 0.684††	0.665	0.311; 1.519††††	1.418	0.419; 3.528††††
	1.0	24 h	**0.134**	**0.031; 0.384**	**0.184**	**0.095; 0.332**	**0.752**	**0.291; 2.041†† ** §§**
		7 d	0.552	0.065; 3.944†††	1.074	0.648; 3.867††††	1.107	0.319; 1.783††††
IL1β	0.1	24 h	**0.033**	**0.028; 0.498**	**0.027**	**0.011; 0.046**	**0.485**	**0.055; 1.139††† * §§§**
		7 d	**0.030**	**0.025; 0.136††**	**0.018**	**0.010; 0.040#**	**0.262**	**0.047; 0.847†† §§**
	1.0	24 h	**0.037**	**0.026; 2.469**	**0.032**	**0.017; 0.041**	**1.920**	**0.058; 5.066††† * §§§**
		7 d	**0.028**	**0.024; 2.002**	**0.013**	**0.009; 0.034#**	**1.795**	**0.036; 4.486††† * §§§§**
IL-6	0.1	24 h	**0.570**	**0.330; 7.022**	**0.315**	**0.175; 0.663**	**10.12**	**0.992; 46.49††† * §§§§**
		7 d	**1.043**	**0.311; 4.756††**	**0.361**	**0.256; 1.461†**	**14.85**	**1.000; 73.66†††† * §§§**
	1.0	24 h	**0.685**	**0.523; 7.521†**	**0.428**	**0.206; 0.793**	**12.77**	**1.295; 38.54††† ** §§§§**
		7 d	**1.131**	**0.561; 12.20†††**	**0.497**	**0.291; 1.387**	**16.65**	**0.932; 61.82†††† §§§§**
IL-8	0.1	24 h	**53.56**	**30.35; 110.4**	**53.14**	**23.75; 75.43**	**147.7**	**59.56; 543.7††† §**
		7 d	276.0	120.9; 1105††	159.4	102.9; 353.8	642.3	93.45; 1550†††
	1.0	24 h	**53.32**	**36.97; 140.6**	**50.84**	**25.50; 70.47**	**101.0**	**60.96; 276.3† §**
		7 d	405.3	120.3; 679.5††	186.5	93.98; 328.3	284.1	78.80; 878.5††
TNFα	0.1	24 h	**0.556**	**0.207; 1.434††**	**0.146**	**0.095; 0.267##**	**1.940**	**0.298; 4.750††† §§§**
		7 d	**0.251**	**0.192; 0.316**	**0.134**	**0.098; 0.180##**	**0.181**	**0.097; 0.257***
	1.0	24 h	**0.446**	**0.196; 7.181†**	**0.155**	**0.106; 0.200##**	**0.955**	**0.476; 13.22††† §§§§**
		7 d	**0.225**	**0.145; 0.732**	**0.085**	**0.065; 0.122###**	**0.278**	**0.124; 0.471†† §§§**

Cells were cultured for the indicated time in the presence of RSV at the indicated multiplicities of infections (MOIs). Cytokine concentrations were measured in supernatants by ELISA. Data were compared to baseline levels (table 3) with Mann Whitney tests: †p<0.05, ††p<0.01, †††p<0.001, ††††p<0.0001; Data were compared between the groups with Kruskal-Wallis test (bold, p<0.05) and post hoc Benjamini Hochberg correction. COPD vs. NS: *p<0.05, **p<0.01, ***p<0.001; COPD vs. S: §p<0.05, §§p<0.01, §§§p<0.001, §§§§ p<0.0001; S vs. NS: #p<0.05, ##p<0.01; ###p<0.01. NS, never smoker, S, current tobacco cigarette smoker; COPD, chronic obstructive pulmonary disease
